# Addressing variability in iPSC-derived models of human disease: guidelines to promote reproducibility

**DOI:** 10.1242/dmm.042317

**Published:** 2020-01-17

**Authors:** Viola Volpato, Caleb Webber

**Affiliations:** UK Dementia Research Institute at Cardiff University, Division of Psychological Medicine and Clinical Neuroscience, Haydn Ellis Building, Maindy Rd, Cardiff CF24 4HQ, UK

**Keywords:** Bioinformatics, Cellular heterogeneity, Reproducibility, iPSCs

## Abstract

Induced pluripotent stem cell (iPSC) technologies have provided *in vitro* models of inaccessible human cell types, yielding new insights into disease mechanisms especially for neurological disorders. However, without due consideration, the thousands of new human iPSC lines generated in the past decade will inevitably affect the reproducibility of iPSC-based experiments. Differences between donor individuals, genetic stability and experimental variability contribute to iPSC model variation by impacting differentiation potency, cellular heterogeneity, morphology, and transcript and protein abundance. Such effects will confound reproducible disease modelling in the absence of appropriate strategies. In this Review, we explore the causes and effects of iPSC heterogeneity, and propose approaches to detect and account for experimental variation between studies, or even exploit it for deeper biological insight.

## Abstract (precis)

**Summary:** This Review discusses approaches to deal with and reduce cellular heterogeneity in induced pluripotent stem cell-based studies, enabling experiments to be reproducible and meaningful.

## Introduction

**Fig. 1.**
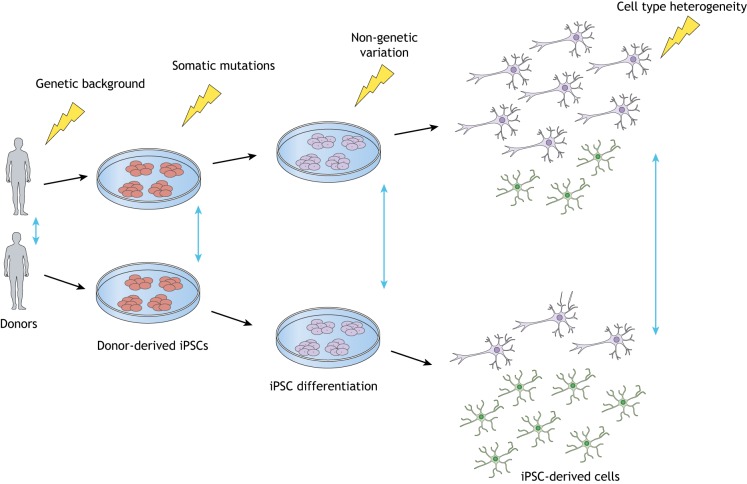
**Variation occurs at each step in an iPSC-based study.** The vertical blue arrows indicate amplification of heterogeneity (Box 1) due to variation (indicated by lightning bolts) created in the previous steps of iPSC derivation.

Since their invention just over a decade ago, induced pluripotent stem cell (iPSC; see Glossary, Box 1)-based models have established a new field in disease modelling, especially for neurological disorders for which inadequate preclinical animal models and poor access to human primary tissue are limiting progress. Although mice, fly or worm models are usually generated within a small number of well-studied genetic backgrounds, thousands of new human iPSC lines have been generated in the UK alone in the past 5 years, each influenced by its unique genetic background (Box 1) and with the vast majority individually receiving very little study. Indeed, the novelty of new genetically interesting iPSC models may be discouraging further study of existing models. Inevitably, differences between donor individuals have been found to affect most iPSC cellular traits, from DNA methylation, mRNA and protein abundance to pluripotency, differentiation and cell morphology (Kilpinen et al., 2017). High variability in differentiation potential and genetic stability between iPSC lines remain subjects of intense research (Guhr et al., 2018). Moreover, even after controlling for genotype, substantial experimental heterogeneity remains. While anatomically matched cell types between two genetically identical animal models might differ little, attempts at experimental replication of iPSC models are thwarted by variation in the derived differentiated cells, and these technical artefacts obscure the biological variation of interest (Volpato et al., 2018). As iPSC culture and differentiation are a multistep process, small variations at each step can inevitably accumulate, generating significantly different outcomes (Fig. 1) (Popp et al., 2018).

**Box 1. Glossary**

**Cellular heterogeneity:** cell type diversity within the experimental cellular population, i.e. due to the presence of multiple cell types and to diversity in morphology, maturation and functionality within each cell type present.

**Expression quantitative trait-associated loci (eQTLs):** genetic variants that are associated with changes in the expression of a gene.

**Genetic background:** the entire set of genes in a genome.

**Genome-wide association studies (GWAS):** hypothesis-free methods that identify associations between genetic loci and phenotypic traits.

**Induced pluripotent stem cells (iPSCs):** stem cells that are generated by induced reprogramming of somatic cells through the forced expression of transcription factors.

**Induced reprogramming:** briefly, it consists of induction of proliferation and downregulation of cell type-specific transcription in a first step, followed by continuous expression of key transcription factors until the iPSC state is established.

**Isogenic iPSC lines:** lines derived from the same individual that are engineered to differ at only one specific locus and are otherwise genetically identical.

**Penetrant genetic variant:** a disease-causing mutation that will cause disease symptoms across most of the individuals carrying it.

**Polygenic risk:** disease risk given by the combined contribution of multiple genetic variants, each variant often of small individual effect.

**Principal component analysis:** a statistical approach that uses orthogonal transformation to identify a set of principal components, each successive linear component capturing as much of the variation in the data as possible.

**Probabilistic estimation of expression residuals (PEER):** based on factor analysis*,* PEER takes as input transcript profiles and covariates from a set of individuals and outputs hidden factors that explain much of the expression variability.

**Removal of unwanted variation (RUV):** a normalisation method that identifies and removes unwanted sources of variation within omics readouts.

**Rosetta line:** an iPSC line that is commonly used within all experiments by multiple laboratories, and that enables researchers to address experimental variation between those laboratories' results.

**Somatic mutations:** acquired (not inherited) genetic alterations that either pre-exist in the somatic cells or can be acquired in the handling of the cells during reprogramming over culture.

**Subclonal:** a mutation that is present in only a fraction of the cells within a population.

The field needs rigorous and well-documented quality control (QC) measures, ‘gold standard’ iPSC lines and standardised protocols, as well as robust statistical analysis to ensure that the information obtained from these efforts are reproducible and meaningful. Reproducibility is a cornerstone of scientific knowledge and, without greater efforts to make iPSC-derived model experiments comparable, this community will be highly vulnerable to error. The aim of this Review is, therefore, to summarise the variables affecting iPSC reproducibility and to propose strategies for each stage of the multistep process to overcome these challenges, thereby enabling experiments to be more readily compared.

## iPSCs as disease models

iPSCs were initially used to model diseases with highly penetrant genetic variants (Box 1) of large phenotypic effect (Cao et al., 2016; Liu et al., 2011; Wainger et al., 2014), but more recently they have been used to study common genetic variants of modest effect size that drive complex diseases. They provide a key platform to study the impact of human cell type-specific gene regulation, as they can recapitulate the broad regulatory profile of their *in vivo* counterparts and also mirror tissue-specific functional genetic variation (Banovich et al., 2018; Santos et al., 2017). Moreover, large-scale iPSC-based studies have identified expression quantitative trait-associated loci (eQTLs; Box 1) that inform on the interpretation of variants identified by genome-wide association studies (GWAS; Box 1) (Carcamo-Orive et al., 2017), as well as protein quantitative trait loci that give insights into mechanisms through which disease-associated genetic risk modulates cell physiology (Mirauta et al., 2018 preprint).

Although the majority of current iPSC differentiation protocols produce immature or fetal-like cells (Handel et al., 2016; Sloan et al., 2017; Yao et al., 2017; Volpato et al., 2018), these cells nonetheless demonstrate a range of cell type-specific characteristics. For example, iPSC-derived neurons are still capable of fundamental neuronal functions, including firing action potentials and releasing neurotransmitters (Bardy et al., 2016). Furthermore, although their maturity might be far from the biological age of disease onset, and they may not display disease-associated cellular phenotypes, researchers have argued that the presence of novel phenotypes in iPSC-derived fetal-like cell models of disease supports ideas that pathologies start long before clinical symptoms appear (Taoufik et al., 2018). This can make iPSC-based models helpful not only in understanding disease mechanisms but also in targeting pre-symptomatic phases of disease. Coenzyme Q10 (Cooper et al., 2012), rapamycin (Cooper et al., 2012), clioquinol (Sandor et al., 2017), tasquinimod (Lang et al., 2019) and the LRRK2 kinase inhibitor GW5074 (Cooper et al., 2012) are all examples of compounds able to rescue disease-associated phenotypes and cell dysfunctions that have been investigated in iPSC-derived dopaminergic neurons (DaNs) from Parkinson's disease patients. iPSC models can also be used in personalised medicine, as demonstrated by the observation that lithium only rescues hyperexcitability phenotypes in neuronal models derived from lithium-responsive bipolar disorder patients, but not in those from non-responsive patients (Mertens et al., 2015).

In pursuit of more accurate modelling of human tissue, co-culture systems consisting of multiple iPSC-derived disease-affected cell types (Zhao et al., 2017; Shi et al., 2013; Odawara et al., 2014), three-dimensional (3D) cultures (Choi et al., 2014) and 3D co-culture organoids (Skardal et al., 2016) have recently been used to recapitulate tissue-level and organ-level dysfunction, whereby the pathology progresses through the interactions between different cell types. However, although these approaches can ameliorate some of the drawbacks of iPSC-based models such as reduced cell maturity, incomplete disease phenotypes and line-to-line variation (Ghaffari et al., 2018), these limitations still need to be effectively addressed to be able to work with patient-derived cells in a high-standard, reproducible and controlled environment. In summary, the promise of iPSC-based human models is clear and the excitement justified. However, in our haste to develop new human cell models, we cannot overlook the fundamental scientific tenet of reproducibility, and the variability within these models is significant.

## Sources and effects of variation in iPSC cultures

iPSC derivation and differentiation are multistep processes and thus small variations at each step can accumulate and generate significantly different outcomes (Fig. 1) (Popp et al., 2018). The substantial impact on the resulting differentiated cells can overwhelm any biological variation of interest, especially where effect sizes are small (Ghaffari et al., 2018).

### Genetic background

It has been widely reported that heterogeneity at the iPSC stage is mainly driven by the genetic background of the donor, more than by any other non-genetic factor, such as culture conditions, passage and sex (Burrows et al., 2016; Kilpinen et al., 2017; Kyttälä et al., 2016). For example, through a systematic generation and phenotyping of hundreds of iPSC lines, the Human Induced Pluripotent Stem Cells Initiative (HipSci) reported that 5-46% of the variation in iPSC cell phenotypes is mainly due to inter-individual differences (Kilpinen et al., 2017). Several other studies have found that iPSC lines derived from the same individual are more similar to each other than to iPSC lines from different individuals. This was highlighted at different levels with inter-individual variation detected in gene expression and eQTLs (Carcamo-Orive et al., 2017; Rouhani et al., 2014; Thomas et al., 2015), and in DNA methylation (Burrows et al., 2016). Although the process of induced reprogramming (Box 1) is based on erasing the existing epigenetic state of the cell of origin (Bilic et al., 2012; Medvedev et al., 2012), the tissue from which the iPSCs were derived and the retention of specific DNA methylation marks can determine the propensity of a line to differentiate into different cell types (de Boni et al., 2018; Roost et al., 2017). Studies have also confirmed that the individual donor's genetic background and differences in differentiation protocols might, in turn, significantly influence the methylation landscape affecting pluripotency between iPSCs from different donors (de Boni et al., 2018; Kim et al., 2014). Unsurprisingly, analyses have shown a higher inter-donor variability in the gene expression of iPSCs-derived models compared to the primary cells they are intended to model (Schwartzentruber et al., 2018), confirming that induction and differentiation procedures themselves introduce variation. Understanding the effects that the genetic background exerts upon the resulting model is necessary as iPSC-specific eQTLs identified through large-scale studies demonstrate that iPSCs have distinct regulatory gene networks compared to their cells of origin (Carcamo-Orive et al., 2017; DeBoever et al., 2017; Kilpinen et al., 2017). Notably, genes for which expression varies with iPSC variability-associated eQTLs are involved in stem cell maintenance and differentiation efficiency/propensity (Carcamo-Orive et al., 2017; Kilpinen et al., 2017; Yamasaki et al., 2017). Predictably, substantial donor effects on protein expression levels are observed for proteins influencing cell differentiation and cell–cell adhesion (Mirauta et al., 2018 preprint). A large-scale quantitative cell morphology assay found donor contribution of up to 23% to the observed phenotypic variation between iPSCs derived from healthy individuals (Kilpinen et al., 2017), confirming that inter-individual variation has significant effects at different levels of the cellular phenotype.

Beyond the genetic background, donor-specific epigenetics retained after reprogramming influence stem cell variation. In particular, the donor-specific background can modulate the Polycomb transcriptional repressors controlling cell identity and development (Carcamo-Orive et al., 2017), and accounts for a significant fraction of inter- and intra-individual iPSC line variability.

### Somatic mutations

Although neither donor age, ethnicity nor sex appear to influence the number of mutations within iPSC lines, ultraviolet (UV)-associated mutations are a major contributor to the heterogeneity in mutation rates across iPSC lines. Thus, source tissue UV exposure will influence the somatic mutation (Box 1) rate (D'Antonio et al., 2018). Reprogramming processes and culturing are thought to influence the selection of somatic variants, notably those associated with cancer, that might be advantageous within the culturing process (Merkle et al., 2017). While variants advantageous to cell culture will increase in frequency (Merkle et al., 2017), it has been reported that 11% of all iPSC somatic variants are subclonal (Box 1) (D'Antonio et al., 2018). Given their frequencies (10-30%), these variants likely arose within the first few cellular divisions after induced reprogramming of the parental cell, which suggests that a single line can contain multiple subclones with altered genetic backgrounds. Compared with clonal variants, subclonal variants showed an enrichment in active promoters and an increased association with altered gene expression (D'Antonio et al., 2018). One of the most recurrent genomic variations found in stem cell cultures is copy number gain of 20q11.21, which is present in up to 25% of embryonic stem cells/iPSCs and affects the differentiation potential of iPSCs (Nguyen et al., 2014). Notably, even when expression changes are not directly detected in iPSCs, variants could have effects in specific differentiated cell types, i.e. mutations in a cardiac-specific transcription factor might affect phenotypes in iPSC-derived cardiomyocytes, but not in iPSC-derived neurons or in the iPSCs themselves (D'Antonio et al., 2018).

### Non-genetic variation

Lastly, several groups reported that variation in routine cell culturing and maintenance such as variation in passage number, growth rate and culture medium contribute to iPSC variability (Fossati et al., 2016; Hu et al., 2010; Schwartzentruber et al., 2018; Volpato et al., 2018), and that automated platforms can reduce such variability (Paull et al., 2015). Our own group has also recently shown that laboratory-based sources of variation, even when different laboratories follow standardised protocols, can substantially overpower genotypic effects. When comparing the transcriptomic readouts of neurons derived from the same iPSC lines following the same protocols across five distinct laboratories, the laboratory of origin accounted for up to 60% of the captured variation. Among its main contributors were passage number and use of frozen progenitors (Volpato et al., 2018). The resulting variation was largely due to different proportions of differentiated cell types within the cultures, despite following the same protocol with the same iPSC lines. Multiple sources of experimental variation contribute, and each contribution is potentially amplified at multiple stages of the culturing process, producing highly heterogeneous cell populations. Along with hindering reproducibility, this heterogeneity can, especially in subsequent omics analyses, mask important biological differences.

## Strategies to reduce variation

**Fig. 2.**
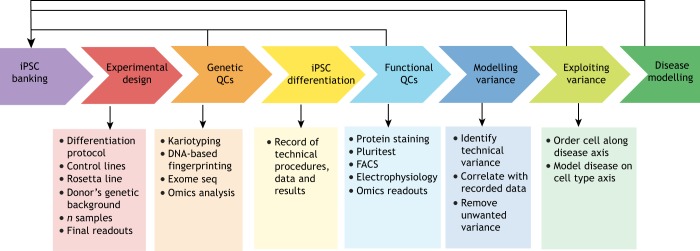
**Flow chart illustrating the approaches to reduce experimental bias and noise.** Experiments should be characterised at each step, from the initial reprogramming and differentiation to the final observation of a disease phenotype. This chart can guide investigators in choosing the most appropriate cell lines and protocols to model a specific disease. Exome seq, whole-exome sequencing; FACS, fluorescence-activated cell sorting; QCs, quality controls.

Acknowledging, measuring and reducing experimental variability must be part of the experimental design. We agree with others that the use of standardised methodologies, along with the documentation of fate, yield and purity of the derived cell types, would increase the reliability of phenotype comparisons and thus improve reproducibility across different laboratories (Engle et al., 2018; Hollingsworth et al., 2017). Fig. 2 illustrates the approaches to reduce experimental bias and noise at each step within an iPSC-based study. As we discussed above, significant variation arises even when researchers closely follow similar protocols and, regardless, protocols may be changed to facilitate new discovery. We thus present strategies that facilitate comparisons across diverse protocols.

### Stem cell banks and reference panels: the need for well-understood lines and common controls

Several large-scale consortia have generated large banks totalling over a thousand iPSC lines and have made these lines available to the research community, including Stem Cells for Biological Assays of Novel Drugs and Predictive Toxicology (StemBANCC) (Cader et al., 2019), HipSci (Leha et al., 2016), the European Bank for Induced Pluripotent Stem Cells (EBiSC) (De Sousa et al., 2017), the iPSC Collection for Omic Research (iPSCORE) (Panopoulos et al., 2017) and others. These iPSC panels offer several advantages; for example, through their systematic creation, curation and QC. The consortia apply rigorous characterisation procedures to examine genomic integrity and filter out lines that harbour somatic variation that might influence cell behaviours (Engle et al., 2018; Popp et al., 2018). Moreover, these lines are often accompanied by whole-exome or genome sequencing data and are subject to extensive transcriptomic and proteomic analyses. The lines available already cover a multitude of disorders, with a particular focus on lines from individuals carrying rare genetic variants (Cader et al., 2019; De Sousa et al., 2017).

Despite these QC advantages, we argue that it is the re-use of lines between studies that will prove key to accounting for variation within these models (Volpato et al., 2018). The genetic background significantly contributes to iPSC cellular heterogeneity (Box 1), including differentiation potency, cellular morphology and gene expression variation (Chandler et al., 2017; Kilpinen et al., 2017). Thus, the effect of a variant of interest must be disentangled from the unique genetic backgrounds of the studied lines. Although the isogenic approaches described below allow researchers to examine the effect of a variant within a specific genetic background, they do not account for the effects of differing genetic backgrounds between different lines across studies. Researchers must also consider that the genetic background in iPSC models could be indirectly affecting the cellular phenotype; for example, by altering the cellular composition of the culture (Volpato et al., 2018). The repeated use of the same or a small number of genetic backgrounds within all other major modelling communities (e.g. mouse, fly, etc) has enabled gene function comparison across studies, empowering systematic genotype/phenotype projects and enabling cross-study knowledge gathering (Doetschman et al., 2009; Smith et al., 2018). Unfortunately, the iPSC modelling community is currently in danger of generating an extensive body of knowledge for which generality is untested and unknown.

The ability of a human iPSC line to model polygenic influence is a unique strength, and the genetic capacity of these models to capture polygenic risk (Box 1) is exciting. Thus, there is a strong argument to vary the genetic background in order to examine the combined contribution of multiple genetic variants towards a phenotype. Moreover, the ability of iPSC models to explore the genetics of any given patient's disorder even without knowledge of any specific disease-causing variant is also a strength. Thus, although useful, we do not argue for the exclusive study of an isogenic bank of lines into which to engineer single variants of interest. Instead, we argue for the inclusion of Rosetta lines (Box 1), a set of case or control lines selected by and shared across each community that can be appropriately re-used across experiments, wherever possible. These Rosetta lines can become the reference points for comparisons across studies, enabling researchers to detect and potentially account for experimental variation between studies (see below). Although studies following exactly the same protocol can obtain significantly different results, the intra-study variation appears to be significantly smaller than the inter-study variation, which suggests a single point of reference for each study (i.e. the inclusion of at least one Rosetta line) might sufficiently represent a large proportion of that study's specific variation. Including multiple Rosetta lines would more accurately capture experimental variation and/or increase the number of comparable studies. Each disease research community might have very different needs for their shared Rosetta lines. For example, the StemBANCC consortium generated a large set of control lines derived from aged controls that could be re-used across studies on a range of neurodegenerative disorders (Cader et al., 2019). Family history or polygenic risk for relevant phenotypic variation within individuals contributing Rosetta lines could also be considered.

### Experimental design

To ensure valid disease modelling experiments, the choice of donors and lines, differentiation protocol, number of samples (both cases and controls) and number of lines from each patient have to be agreed primarily based on the disease to be modelled, the likely effect size of disease-relevant phenotypes and the readouts of interest. The highly detailed and curated iPSC banks and reference panels described above provide excellent starting points to help define a controlled and robust experimental design.

Assuming a straightforward study, such as the comparison of two populations – cases versus controls, we recommend increasing the number of cases and controls rather than increasing the number of lines derived from each case or control individual, as this powers the more interesting population comparison. An analysis of transcriptomic profiles of undifferentiated iPSCs found that four to six individuals per group provided a reasonable balance of sensitivity and specificity (Germain et al., 2017), although this number will vary significantly depending on the genetic effect studied, e.g. smaller effect sizes will require larger numbers. Remarkably, the same study reported that using multiple iPSC lines from the same individual actually increased spurious differences in gene expression between study groups. Nonetheless, having multiple lines available from each individual enables the examination of outliers for line-specific effects (e.g. somatic variation – see above) and validation of key results.

Control lines should be matched for age, sex and ethnicity. Whenever possible, these should also match the time in culture. If female iPSC lines are used, they should be characterized for X-chromosome inactivation status. Considerable variation in the amount of X-chromosome reactivation in early-passage lines and random re-inactivation of X chromosome in later passages affect differentiation potential (Booth et al., 2019; DeBoever et al., 2017; Mekhoubad et al., 2012; Salomonis et al., 2016).

Alternatively, a widely used strategy to deal with genetic background influence on the expression of a disease phenotype in the case of a known genetic variant is to use clustered regularly interspaced short palindromic repeats (CRISPR)/Cas9-mediated genomic editing technologies to generate isogenic cell line (Box 1) pairs (Cong et al., 2013; Yumlu et al., 2017). Although straightforward, this approach is not always ideal. For example, where a particular genetic variant is not sufficient to cause disease, i.e. it is also found in the non-diseased population but at a significantly lower frequency, the genetic background could also be influencing the disease process. If so, examining the risk variant within a patient line, which is more likely to have a risk-increasing or -modifying genetic background, could be key for elucidating its aetiological contribution. Thus, it is usually a better strategy to edit the risk variant out of a patient line than to edit the variant into a control line. However, comparing the effect of editing such a variant out of a disease line to the effect of engineering the variant into a control line would inform on the contribution of the genetic background. Where a variant is apparently highly penetrant (Box 1), it would be better engineered into a well-studied control line.

### Obtaining homogeneous cellular composition

When differentiating iPSCs into the cell type of interest, the cellular composition of the resulting culture is a primary source of inter- and intra-experimental variation (Sandor et al., 2017; Volpato et al., 2018). Although standardised protocols that produce more homogeneous and more mature populations of cell types in a compressed time frame are attractive to reduce variability (Nehme et al., 2018), researchers should take care that the relevant biology is not skipped over and thus missed (Schafer et al., 2019). Rigorous standardisation of iPSC reprogramming and differentiation can preserve inter-individual variation in iPSC-derived differentiated cells with high fidelity and without increasing intra-individual variation, thus reducing the previously reported intra-clone variation (Matsa et al., 2016). Other strategies have proven effective in obtaining more homogeneous iPSC-derived lines through functional QC analyses. Electrophysiological activity assays can increase confidence in cell type specificity and provide experimental readouts that can be used as standards for achieving consistent models across multiple rounds of differentiation (Ghaffari et al., 2018). Isolating the cell type of interest through the expression of marker genes, most conveniently on the cell surface, is an effective strategy for rapid isolation and characterization. However, for certain cell types of interest, most notably neurons, a suitable cell surface marker is not known and currently only neuronal nuclei can be isolated (Matevossian et al., 2008). In another approach, a construct with a tyrosine hydroxylase promoter driving a fluorescent reporter was used to sort fixed iPSC-derived DaNs. Subsequent transcriptomic analysis of the sorted and unsorted lines revealed that 34% of the total transcriptomic variation was attributable to cell type heterogeneity in DaN differentiation protocols and that the cell type purification step increased transcriptomic uniformity in the purified lines (Sandor et al., 2017). However, both nuclei and fluorescence-activated cell sorting of fixed cells allow limited downstream assays, whereas sorting live cells might itself introduce biases (Llufrio et al., 2018).

### Identify and remove unwanted variation

If the gene expression profile is of interest, single-cell RNA sequencing provides an excellent option to identify and distinguish heterogeneous cell populations. Covariation across gene expression profiles identifies shared cell types or states, which can be grouped into individual populations by computational clustering approaches (Kiselev et al., 2019). Clustering cells into distinct populations can identify iPSC-derived cells that best approximate the native cell type for use in subsequent analyses (Paik et al., 2018; Volpato et al., 2018). Machine learning-based methods, trained on large-scale *in vivo* gene expression and/or *in vitro* cellular physiological data, can be used to identify the molecular signatures of different functional states of differentiated cells (La Manno et al., 2016). When such differences in maturation state are recognised, they can be regressed out to reveal the biological variation of interest (Buettner et al., 2015). Similarly, proteomic analyses allow detection of the cells' volume or cell cycle stage by profiling marker protein expression and DNA content, enabling normalisation (Akopyan et al., 2014; Kafri et al., 2013). Gene expression variation of cell type markers can be also used to estimate cell type heterogeneity in bulk RNA-sequencing experiments. If the cell types within a culture have been previously well characterised, deconvolution approaches will use these profiles to estimate the cell type proportions within a heterogeneous culture (Wang et al., 2019; Zaitsev et al., 2019). However, single-marker genes can also be used. For example, *GFAP* expression correlates well with the proportion of astrocytes within iPSC-derived cultures (Booth et al., 2019). This was subsequently used as a measure of iPSC differentiation efficiency, enabling researchers to disregard uninteresting gene expression variation.

**Fig. 3.**
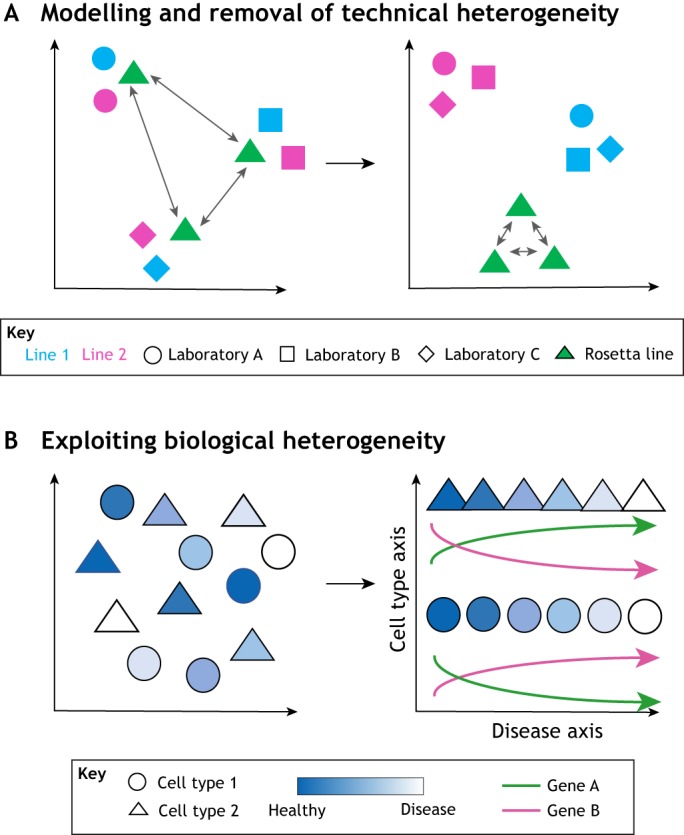
**How to address and exploit heterogeneity to model disease.** The cell lines are plotted on axes that represent the principal dimensions of variation from an omics measurement (e.g. gene expression via RNA sequencing). (A) Identify and remove technical or non-relevant variation between lines by assuming similarity between the same Rosetta line used in the different studies. When variation between the Rosetta line instances is removed using methods such as removal of unwanted variation (RUV), the biological variation between different cell lines can be exposed/unmasked. (B) Use single-cell assays to distinguish cell types and then use within-population heterogeneity to arrange individual cells along a pseudotemporal axis describing progression through a biological process. Each assayed cell provides a stepping stone through the process of interest and the changes in the expression of individual genes along this process can be inferred.

When the causes of variation are unknown and cannot be easily regressed out from the data, factor analysis-based approaches can capture variance between samples that, upon correlation to recorded sources of variation such as experimental confounding factors, can be safely removed from the data. Clearly associating trends in the data with unwanted experimental variation is important in order to justify their removal. Researchers cannot simply ignore trends because their biological meaning is unclear or uninteresting. Methods range from the simple principal component analysis (Box 1) to the more articulate probabilistic estimation of expression residuals (PEER; Box 1) approach ('t Hoen et al., 2013; Vigilante et al., 2019) and generalized linear model based methods such as the removal of unwanted variation (RUV; Box 1) strategy (Risso et al., 2014). For instance, if Rosetta lines are used as a reference point across different studies, RUV could help identify laboratory- and experiment-dependent variation between the omics readouts of Rosetta lines that, in turn, would help unmask the biological variation of interest between the experimental lines across studies (Fig. 3A). Our group has recently applied RUV to model variance according to the experimental design, whereby a laboratory-dependent variation was identifiable assuming similarity between the technical replicates across different laboratories (Volpato et al., 2018). Such tools can incorporate the effects of several types of covariates and, by using negative-control genes or replicate samples, model both technical and biological variability (Carcamo-Orive et al., 2017; Ran et al., 2017; Volpato et al., 2018). Moreover, RUV-based tools can cover a wide spectrum of omics data, from gene expression, proteomics and metabolomics to imaging data (https://statistics.berkeley.edu/sites/default/files/tech-reports/ruv.pdf).

### Exploiting cellular heterogeneity

In culture, cells that are used to model a disease-relevant process are unlikely to be undergoing that process in a highly synchronised manner. Taking a single measurement of a property of the culture cannot capture this heterogeneity. Instead, it captures cells at various points in the process, summed into a single value that usually does not recapitulate the modelled process in a helpful way. However, single-cell transcriptomics allows researchers to distinguish cells that are undergoing distinct processes or are at different stages within the same process. The presence of distinct cellular processes between patient-derived cell models can identify distinct aetiologies between patients, which can prompt clinical re-evaluation, even leading to a different diagnosis (Lang et al., 2019).

For cells undergoing the same biological process, pseudotemporal ordering approaches attempt to arrange the cells based on their progression through that process (Fig. 3B). Reasoning that cells with more similar gene expression profiles are more likely to be at a similar stage in the process than cells with less similar gene expression profiles, cells can be ordered to generate a pseudotemporal profile within the modelled cellular process. In effect, each cell provides a snapshot of the unfolding disease process with similar snapshots (gene expression profiles) placed closer together within the series to provide a continuum across which researchers can infer changes in the expression of individual genes. This technique enables the identification of early gene dysregulation events that, upon correction, can restore later (typically more severe) gene dysregulation and ameliorate disease-related cellular phenotypes (Lang et al., 2019). In addition, as intrinsic properties of iPSC lines can result in varying cell types of different proportions (Volpato et al., 2018), single-cell analyses of cell-type and intra-culture heterogeneity may also reveal unique developmental phenotypes where genetic variants affect cell types, cell type proportions and the resulting cell type circuitries. For instance, when iPSCs carrying genetic variants that cause the neuromuscular disorder metachromatic leukodystrophy are differentiated into heterogeneous mixed populations of oligodendrocytes, neurons and astrocytes, the disease-causing mutation favours the maintenance of immature oligodendroglial progenitors and impairs their differentiation with consequent reduction in neuronal function support and eventually neuron death (Frati et al., 2018). This mechanism has been confirmed to contribute to the early stages of pathology before neurodegeneration occurs, pointing to the importance of such models to investigate disease processes that are shaped early in brain development and cannot be properly assessed in brain tissues of patients at the late stages of the disease, with implications for the timing and efficacy of treatments.

## Future perspectives

iPSC models offer tremendous opportunities to advance our understanding across a wide range of biology. Each model is as unique as the individual from whom it was derived, along with a large amount of known and unknown experimental variation. Although every effort should be made to understand and reduce experimental variation, a more immediate strategy would be for each iPSC modelling community to adopt a set of appropriate common case and control lines that would enable them to identify experimental variation across studies. Through this simple step, bioinformatics approaches can help to identify and remove bias from omics measurements, aiding inter-study comparisons and thus scientific reproducibility. Finally, single-cell assay technologies may provide opportunities not only to reduce study heterogeneity but also to convert process and progressional heterogeneity into significantly insightful biology.
